# Protein Array Profiling of Tic Patient Sera Reveals a Broad Range and Enhanced Immune Response against Group A *Streptococcus* Antigens

**DOI:** 10.1371/journal.pone.0006332

**Published:** 2009-07-22

**Authors:** Mauro Bombaci, Renata Grifantini, Marirosa Mora, Valerio Reguzzi, Roberto Petracca, Eva Meoni, Sergio Balloni, Chiara Zingaretti, Fabiana Falugi, Andrea G. O. Manetti, Immaculada Margarit, James M. Musser, Francesco Cardona, Graziella Orefici, Guido Grandi, Giuliano Bensi

**Affiliations:** 1 Novartis Vaccines and Diagnostics, Siena, Italy; 2 Center for Molecular and Translational Human Infectious Diseases Research, The Methodist Hospital Research Institute, Houston, Texas, United States of America; 3 Department of Child and Adolescent Neuropsychiatry, University La Sapienza, Rome, Italy; 4 Respiratory and Systemic Bacterial Diseases, Istituto Superiore di Sanità, Rome, Italy; Columbia University, United States of America

## Abstract

The human pathogen Group A *Streptococcus* (*Streptococcus pyogenes*, GAS) is widely recognized as a major cause of common pharyngitis as well as of severe invasive diseases and non-suppurative sequelae associated with the existence of GAS antigens eliciting host autoantibodies. It has been proposed that a subset of paediatric disorders characterized by tics and obsessive-compulsive symptoms would exacerbate in association with relapses of GAS-associated pharyngitis. This hypothesis is however still controversial. In the attempt to shed light on the contribution of GAS infections to the onset of neuropsychiatric or behavioral disorders affecting as many as 3% of children and adolescents, we tested the antibody response of tic patient sera to a representative panel of GAS antigens. In particular, 102 recombinant proteins were spotted on nitrocellulose-coated glass slides and probed against 61 sera collected from young patients with typical tic neuropsychiatric symptoms but with no overt GAS infection. Sera from 35 children with neither tic disorder nor overt GAS infection were also analyzed. The protein recognition patterns of these two sera groups were compared with those obtained using 239 sera from children with GAS-associated pharyngitis. This comparative analysis identified 25 antigens recognized by sera of the three patient groups and 21 antigens recognized by tic and pharyngitis sera, but poorly or not recognized by sera from children without tic. Interestingly, these antigens appeared to be, in quantitative terms, more immunogenic in tic than in pharyngitis patients. Additionally, a third group of antigens appeared to be preferentially and specifically recognized by tic sera. These findings provide the first evidence that tic patient sera exhibit immunological profiles typical of individuals who elicited a broad, specific and strong immune response against GAS. This may be relevant in the context of one of the hypothesis proposing that GAS antigen-dependent induction of autoantibodies in susceptible individuals may be involved the occurrence of tic disorders.

## Introduction

Group A *Streptococcus* (*Streptococcus pyogenes*, GAS) is a common human pathogen responsible for a large variety of infections which most frequently occur at the level of the upper respiratory tract and skin causing mild diseases such as pharyngitis, impetigo and cellulitis. Less frequently, GAS infections result into life-threatening and invasive conditions, such as bacteremia, pneumonia, necrotizing fasciitis (NF) and streptococcal toxic shock syndrome (STSS) [1]. Differently from other streptococci, GAS infections can also lead to non suppurative sequelae, such as acute rheumatic fever (RF) and post-streptococcal glomerulonephritis, which appear to be associated with autoimmune reactions due to “molecular mimicry” between host tissues and M protein, the major GAS surface-associated antigen. In fact, anti-M specific antibodies have been proven to cross-react with human tissues, including the heart, skeletal muscle, brain and glomerular basement membranes [2].

Post-infectious disorders secondary to GAS infections, especially RF, may present with a wide array of neurological and psychiatric pictures, characterized by the association of movement disorders (mainly chorea and tics) and behavioral disorders (mainly obsessive-compulsive symptoms, anxiety and mood disorders) [3]. The systematic classification of post-streptococcal neuropsychiatric disorders is still in progress. Two entities are however universally acknowledged: Sydenham's chorea (SC) [4], which constitutes one of the major criteria for the diagnosis of acute RF, and post-streptococcal acute disseminated encephalomyelitis (PSADEM) [5]. In the last 15 years, it has been suggested that the clinical spectrum of these disorders might be broader. Particularly, in 1998 Swedo et al. proposed the existence of a paediatric disorder mainly characterized by tics and obsessive-compulsive symptoms exacerbating in association with relapses of streptococcal pharyngitis [6]. They indicated this phenotype with the acronym PANDAS (Paediatric Autoimmune Neuropsychiatric Disorders Associated with Streptococcal infections). This hypothesis, however, generated significant controversy, one critical issue being represented by the difficulty in confirming a temporal relationship between neuropsychiatric symptoms and GAS infections [7]–[9].

In the attempt to shed some light on the possible contribution of GAS infections to the onset of neuropsychiatric or behavioral disorders, we assessed whether a broad antibody response against GAS antigens could be revealed in young patients with tic disorder, a pathological condition consisting of “sudden, repetitive, non-rhythmic, involuntary movements (motor tic) or sounds (phonic tic) that involve discrete groups of muscles” [10]. Sera obtained from these children were tested for their immunological reactivity versus a representative panel of GAS antigens. The analysis was carried out using the protein array technology [11], which allows high throughput analysis of human sera against a large number of antigens. Protein chips containing 102 GAS proteins were probed with sera from tic patients and children without tics. The resulting data were compared with those obtained with chips probed with GAS-associated pharyngitis patient sera. By using this experimental approach, we were able to better define the relationship between tic disorder and immune response to GAS antigens.

## Results

### Micro array design and validation

**Figure 1:**
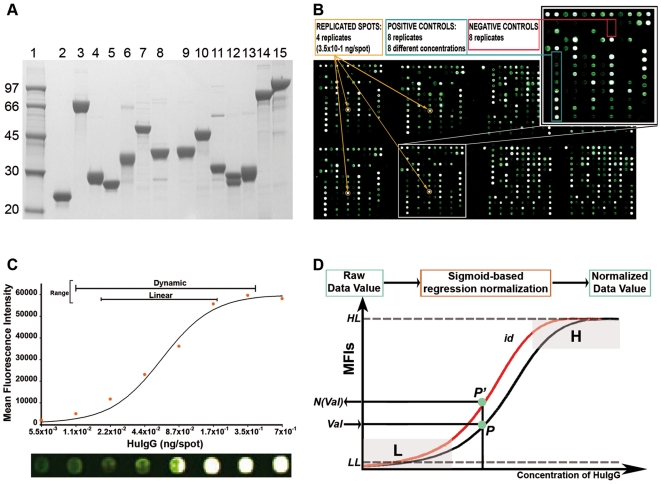
Protein micro array set-up and validation. A, SDS-PAGE analysis of purified recombinant GAS proteins stained with Coomassie. Molecular weight markers in lane 1. B, Representative image of a chip after incubation with a human serum and with Cy3-labelled anti-human IgG and Cy5-labelled anti-human IgM. Replicates of tested antigens and of negative and positive IgG and IgM controls are highlighted. C, graphic representation of the control human IgG curve. Orange dots correspond to the different IgG concentrations measured on the x-axis, while the continuous line corresponds to the interpolated resulting curve. MFIs values are reported on the y-axis. The chip image of different IgG concentration revealed by incubation with anti-human IgG-Cy3 is shown below the graph. D, Sigmoid-derived data normalization method. Data are normalized using the sigmoid control curve (black) referred to a reference sigmoid curve (red). IgG control concentrations and MFIs are reported on the x- and the y-axis respectively. HL, high level signal area (normalized MFI value >40,000); LL, low level signal area (normalized MFI value <15,000); id, ideal sigmoid curve; P and P', intersection points of not normalized and normalized MFI values on the experimental and reference sigmoid curve; Val and N (Val): Background-subtracted MFI value and normalized value resulting after normalization, respectively.

To study the serological response of tic patient versus a representative panel of GAS antigens a protein array was generated by printing 102 recombinant proteins, mainly selected from the GAS SF370 M1 genome (Table S1, Supplementary Information). The majority of printed GAS proteins were expressed as C-terminal His-tag fusions while 23 proteins where expressed as double fusions, with glutathione S-transferase (GST) at the N-terminal and with a His-tag at the C-terminal. Proteins obtained after affinity purification from the bacterial soluble fraction showed purity levels equal or greater than 70%, as estimated by densitometric scan of PAGE-SDS gels (Fig. 1A). The protein array validation was obtained by using a defined printing scheme and control spots (Figure 1B). Printing 4 replicates of each antigen followed by incubation with mouse anti-sera raised against the recombinant proteins and/or GST and His fusion tags, assured that all of them were efficiently and reproducibly immobilized on nitrocellulose slides (not shown). PBS buffer, spotted on either side of each protein spot was used to detect protein carry-over during spotting and fewer than 10% of PBS spots showed signal intensities higher than the average nitrocellulose background fluorescence intensity (FI) value (see Materials and Methods). Proteins eluted after affinity purification of total soluble extracts from an *E. coli* strain carrying the empty expression vectors were also printed on the array to determine the maximum background signal due to contaminants possibly generated by incubation with the sera under investigation, which always resulted in a mean fluorescence intensity (MFI) not higher than 1023 (Standard deviation, SD, 461). Finally, 8 different amounts of human IgGs were spotted on the array in 4 replicates (from 6×10^−3^ to 7×10^−1^ ng of immobilized protein per spot) and used as controls for detection, system reproducibility and data normalization. MFIs of human IgG spots, obtained after detection with Cy3-conjugated anti-human antibodies, were best fitted by sigmoid curves showing a signal dynamic range of about 2 logs and, within this range, a linearity covering approximately 1 log of FI values, with a lower detection limit which resulted to be approximately 7×10^−3^ ng (Figure 1C). To compare data from different experiments we used a normalization method previously set up and validated for our system (Reguzzi V., personal communication), in which the experimental IgG curve of each slide was adjusted on a reference sigmoid IgG curve, and the background-subtracted MFI values of each protein were normalized accordingly (Figure 1D and Materials and Methods). The definition of the MFI of the reference IgG curve also permitted us to assess that the intra-slide coefficients of variation (CV) was lower than 5%, while the inter-slide CV, measured by computation of human IgG MFI values of 50 slides, ranged from 46% to 30% for FI values lower than 15,000 (value corresponding to the normalized MFI value of buffer spots plus 2 standard deviations as described in Materials and Methods) and decreased to approximately 20% for FI values higher than 15,000. On the basis of these results, a normalized FI value of 15,000 was arbitrarily chosen as the lowest signal threshold for scoring a protein as positively recognized by human sera. In addition, a second FI cut off of 40,000 was arbitrarily defined to mark highly reactive proteins and/or identify high titer sera, which in our experimental system corresponded approximately to the saturation segment of the human IgG curve (Figure 1D).

### Serological profiling of tic, no tic and pharyngitis sera

**Figure 2:**
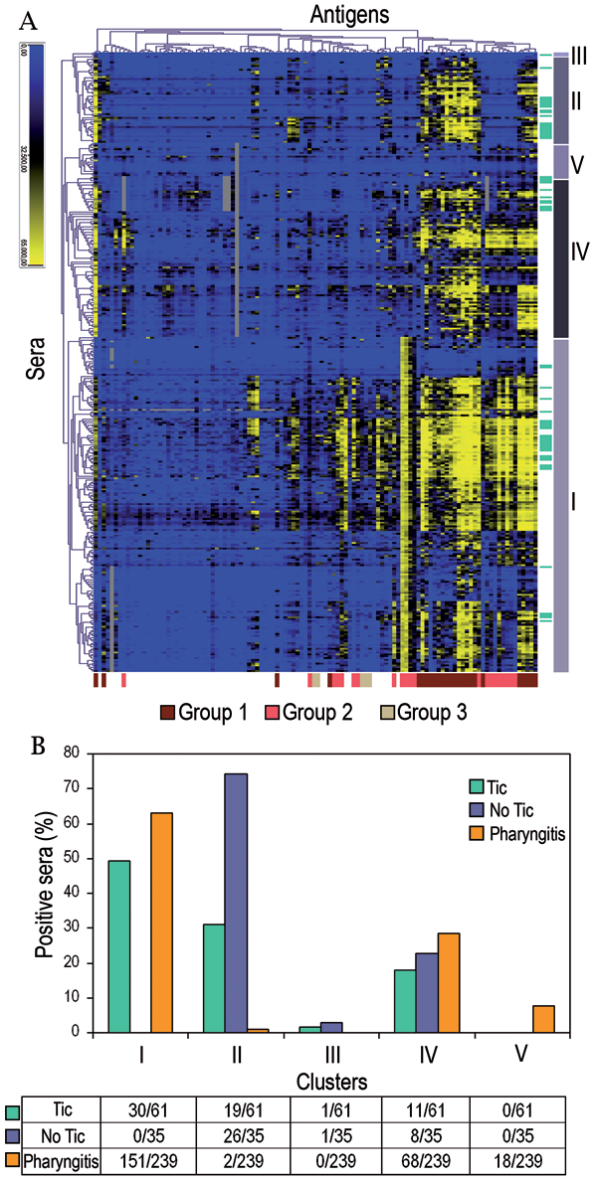
Tic, No Tic and Pharyngitis sera analysis. A, Unsupervised hierarchical clustering of human Tic (*n* = 61), No Tic (*n* = 35) and Pharyngitis (*n* = 239) sera versus the 102 selected GAS antigens. Antigens/sera interactions resulting in signals with high or low FI are visualized in yellow and blue respectively. Color scale of signal intensity is reported on top-left. Clusters group antigens and/or sera showing similar reactivity profiles. Bars and roman numbers on the right of the dendrogram identify the sera clusters considered for the analysis. Light green lines next to cluster bars highlight how the 61 tic sera distribute within the dendrogram. Colored bars at the bottom of the dendrogram highlight the distribution of group 1, group 2 and group 3 antigens, as defined in Tables I, II and III. B, Distribution of sera in the different clusters. The histogram shows the percent of positive sera (FI>15,000) for each class in relation to the five defined clusters. The number of positive sera in each cluster calculated on the total number of sera tested for each group is shown in the table below the histogram.

In the attempt to determine if there is a link between GAS infection or exposure to GAS antigens and tic disorders, the GAS protein array was probed with 61 sera from patients with tics. At the time of the visit patients did not show clinical signs of pharyngitis and the percentage of GAS carriers in the group (14.7%) did not differ from that observed in a normal children population falling in the same age range [12]. A similar analysis was carried out using 35 sera from children without tic disorder and without pharyngitis symptoms (referred to as “no tic”) and 239 sera from GAS-pharyngitis patients (confirmed by throat swab and isolation of the infective GAS strain). All groups were formed by 4–14-year-old children. Sera reactivity was evaluated by detecting total IgG bound to each protein spot using fluorescently labeled anti-human IgG and measuring the FI values for each antigen. To define the antigen recognition pattern of the three sera groups, the normalized FI values were subjected to unsupervised bi-dimensional hierarchical clustering using the Pearson algorithm to calculate cluster distances. The clustered view of the antibody recognition profiles of the GAS antigens observed for tic, no tic and pharyngitis patients, for a total of 335 sera, is shown in Figure 2A. This analysis resulted in the definition of 5 different major clusters at the second level of hierarchy. Cluster I included 181 sera (30 tic and 151 pharyngitis), cluster II 47 sera (19 tic, 26 no tic and 2 pharyngitis), cluster IV 87 sera (11 tic, 8 no tic and 68 pharyngitis). Clusters III (1 tic and 1 no tic) and V (18 pharyngitis) included a minority of the tested sera. Most of the tic sera distributed without any statistically significant difference in clusters I, II and IV, while pharyngitis sera were grouped in clusters I, IV and V (Figure 2B). Remarkably, nearly 80% of the no tic sera segregated in cluster II, indicating that the large majority of sera belonging to the negative control group had a significantly different antigen recognition pattern as compared to the other two sera classes.

### Specific immunogenicity of GAS antigens in tic, no tic and pharyngitis patients

When we analyzed the tic sera reactivity frequencies for each single antigen, 50% of them (51 antigens) resulted to be recognized by at least 30% of the tic patient sera (18 sera), while approximately 25% resulted to be reactive against less than 10% of them. Three groups of immunogenic antigens could be distinguished: (i) one consisting of 25 proteins (Table 1), which were recognized with similar frequencies by all three sera families. Several known immunogenic GAS antigens fell in this subset, such as M proteins, streptolysin O (SLO), streptokinase A and C5a peptidase precursors. The fact that these antigens were also recognized by sera belonging to the no tic negative control group, was not considered contradictory since these individuals had most probably already experienced GAS infections; (ii) another group of 21 proteins (Table 2), recognized with comparable frequencies by both tic and pharyngitis sera but with statistically significant lower frequencies by no tic sera; (iii) a third group of 5 proteins (Table 3 and Figure S1, Supplementary Information), which were instead recognized with statistically significant higher recognition rates by tic sera compared to both pharyngitis and no tic sera. Interestingly, approximately 40% of tic sera (24 out of 61 tested) were simultaneously reactive against at least 3 of these antigens (Table 4).

**Table 1:** GAS antigens reacting against tic, pharyngitis and no tic sera.

SPy(a)	Annotation	Tic	Phar (b)	No Tic
SPy0269	putative surface exclusion protein	98	89	93
gi-507127	M protein type 9	97	87	89
SPy2010	C5A peptidase precursor	92	67	89
MGAS10270_SPy1784	M protein type 2	89	94	83
SPy1801	immunogenic secreted protein precursor homolog	89	84	83
SPy1813	hypothetical protein	89	82	94
SPy1979	streptokinase A precursor	89	81	94
SPy1361	putative internalin A precursor	89	80	91
gi-126660	M protein type 12	89	66	86
SPy0167	streptolysin O precursor	89	63	97
SPy0843	hypothetical protein	87	81	74
SPy0857	putative peptidoglycan hydrolase	87	76	94
gi-4586375	M protein type 23	87	74	83
SPy2025	immunogenic secreted protein precursor	87	59	89
SPyM3_1727	M protein type 3	85	83	86
SPy0416	putative cell envelope proteinase	85	62	83
SPy2043	mitogenic factor	82	59	91
SPy2018	M protein type 1	74	85	63
SPy0019	putative secreted protein	74	49	83
SPy1972	putative pullulanase	70	50	60
SPy0457	putative cyclophillin-type protein	57	46	34
SPy2009	hypothetical protein	46	33	26
SPy1733	putative transcription regulator	43	35	26
SPy0436	putative exotoxin	38	25	31
SPy1007	streptococcal exotoxin I	30	40	43

Numeric values refer to % of positive sera of each class versus each antigen.

(a) When the SPy number is not available, the gi- number is indicated.

(b) Pharyngitis.

**Table 2:** GAS antigens preferentially reacting against tic and pharyngitis sera.

SPy	Annotation	Tic	Phar (a)	No Tic
SPy0747	hypothetical protein	90	92	51
SPy0031	putative choline binding protein	64	83	29
SPy1032	extracellular hyaluronate lyase	62	74	29
SPy0159	hypothetical protein	59	69	26
SPy0737	putative extracellular matrix binding protein	59	60	17
SPy0793	hypothetical protein	56	65	22
SPy1037	hypothetical protein	54	52	20
SPy0714	putative adhesion protein	54	44	17
SPy2000	surface lipoprotein	52	64	6
SPy1390	putative protease maturation protein	51	77	9
SPy2037	peptidylprolyl isomerase	51	72	9
SPy0252	putative sugar transporter sugar binding lipoprotein	51	69	3
SPy1882	putative acid phosphatase	51	62	9
SPy1983	collagen-like surface protein	51	46	23
M5005_SPy0249(b)	oligopeptidepermease OppA	49	63	20
SPy0287	hypothetical protein	49	60	9
SPy0441	hypothetical protein	43	52	6
SPy2007	putative laminin adhesion	38	27	0
SPy1326	hypothetical protein	38	28	9
SPy2066	putative dipeptidase	33	31	11
SPy0838	hypothetical protein	30	34	4

Numeric values refer to % of positive sera of each class versus each antigen. Recognition percentages from No Tic sera were significantly lower (P<0.05) when compared to those obtained with either Tic or Pharyngitis sera, as established by using the two-tailed χ^2^ test.

(a) Pharyngitis.

(b) M5005 SPy number used since the protein, although present, was not annotated in SF370 M1 strain.

**Table 3:** GAS antigens preferentially reacting against tic sera.

SPy	Annotation	Tic	Phar (a)	No Tic
SPy0453	metal binding protein of ABC transporter	41	25	9
SPy1939	hypothetical protein	39	13	11
SPy1795	putative ABC transporter	38	22	3
SPy1054	putative collagen-like protein	36	14	3
SPy1306	maltose/maltodextrin-binding protein	34	15	0

Numeric values refer to % of positive sera of each class versus each antigen.

Number of tic, pharyngitis and no tic sera positive against each antigen were compared using two-tailed χ^2^ test, resulting in a statistically higher number of positive tic sera as compared to pharyngitis and no tic sera (P<0.05).

(a) Pharyngitis.

**Table 4:** Sera reacting against multiple tic-preferentially-recognized antigens.

Number positive antigens(a)	Tic(b)	Pharyngitis(b)	P value(c)	No Tic(b)	P value(d)
5	23% (14/61)	5% (11/239)	<0.0001	0% (0/35)	0.0057
at least 4	33% (20/61)	6% (15/239)	<0.0001	0% (0/35)	0.0004
at least 3	39% (24/61)	17% (41/239)	0.0003	3% (1/35)	0.0002

(a) number of antigens reported in Table 3 simultaneously recognized by the same serum.

(b) percentages of positive sera (number of positive sera/total sera tested) simultaneously recognizing the number of antigens indicated in the first column.

(c) P values of Tic versus Pharyngitis calculated with two-tailed χ2 test.

(d) P values of Tic versus No Tic calculated with two-tailed χ2 test.

The possibility that a correlation existed between the observed serological profiles and an ongoing or recent GAS infection was evaluated by considering the anti streptolysin O (ASO) titers of tic patients. According to our previous works [13] we fixed the cut-off of 407 International Units (I.U.) to define high or low ASO titer. When 31 tic sera with high ASO were compared to 16 tic sera with low ASO, we did not observe major differences neither in the type nor in the total number of recognized GAS antigens (not shown).

**Figure 3:**
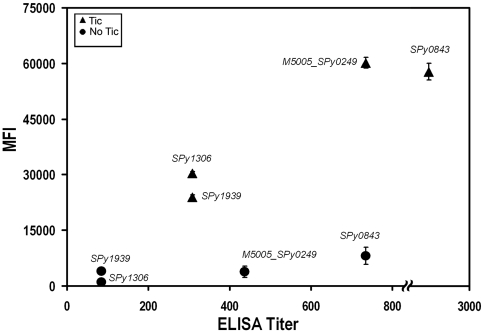
Correlation between sera ELISA titers and MFI. Black circles and triangles represent the ELISA Titers (geometric means, x-axis) and Mean Fluorescent Intensities (arithmetic means, y-axis) obtained using 8 sera from either no tic or tic patients with each of the antigens Spy0843, M5005_Spy0249, Spy1306 and Spy1939. Standard errors of the means are reported. Both ELISA titers and MFIs differences between the two sera classes were statistically significant with P values<0.05 calculated either with T Student (ELISA) or Fisher's exact test (MFI).

To confirm and further validate our data, IgG levels against few test antigens included in the three groups were analyzed by standard methods. When 8 negative sera (MFI<15,000) and 8 positive sera (MFI>15,000) against each of the antigens Spy0843, M5005_Spy0249, Spy 1306 and Spy 1939 were tested by ELISA, statistically significant titer differences were observed among negative and positive sera, as defined on the basis of the MFIs resulting from the micro-array analysis (Figure 3).

### Comparison of the immunoreactivity of tic sera

**Figure 4:**
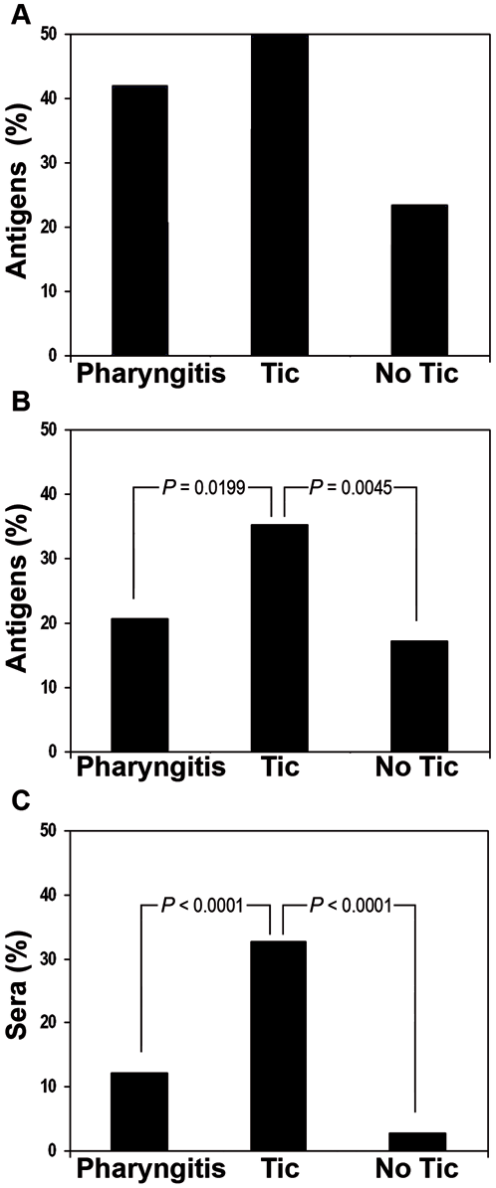
Relative reactivity of the three sera classes. A, Percentages of tested GAS antigens recognized by at least 30% of sera for each patient group. B, Percentages of antigens recognized by at least 30% of the sera of each class with FI>40,000. C, Percentages of sera for each class reacting with at least 30% of the antigens and with FI>40,000. Numbers above histograms bars indicate P values calculated with the two-tailed χ2 test.

The relationship between tic and anti-GAS immune response was confirmed and strengthened by comparing the overall reactivity of the three sera families against the 102 antigens spotted on the chip. While the percentages of antigens recognized by >30% of tested sera (>18 tic, >72 pharyngitis and >10 no tic sera) were not remarkably different for the tic and pharyngitis groups (51 and 43 antigens respectively), no tic sera recognized overall fewer antigens (24 antigens) (Figure 4A). Significant divergences between tic sera and the other two sera families were instead observed when the differences in spot intensities were considered, as indicative of differential antigen-specific IgG levels. In fact, we verified which were the percentages of antigens recognized most intensely by the three sera families (FI>40,000, arbitrary cut-off) and, conversely, which the percentages of sera generating on the array a very high signal (FI>40,000) against at least 30% of the tested antigens (31 antigens). The percentage of GAS antigens recognized by the tested sera with FI>40,000 was 36% in the case of tic patients (37 antigens), 21% in the case of pharyngitis patients (21 antigens) and 18% (18 antigens) in the no tic group (Figure 4B). Similarly, 33% of the tic patient sera (20 sera) reacted very intensively (FI>40,000) with at least 30% of the spotted antigens (31 antigens), as opposite to 12% of the pharyngitis patient sera (29 sera) and only 3% (1 serum) of the negative control group sera (Figure 4C). In both cases, statistical analysis carried out using the χ2 test and referring to a P value<0.05 confirmed that the differences observed between the reactivity of tic sera and the other two sera groups were statistically significant.

## Discussion

In this work we have addressed the issue of whether a consistent and strong correlation between tic disorders and GAS infection could be established. To this purpose, we have carried out the first systematic analysis of the IgG response of tic patients sera versus a representative panel of GAS antigens and the resulting data were compared to those obtained with sera from children either without tic or with pharyngitis.

The protein micro array approach which we exploited turned up to be appropriate to test a total of almost 350 human sera against over 100 bacterial antigens, demonstrating that this technology should be taken into account whenever large sets of data on *in vivo* expression of pathogen antigens and on the subsequent host immune response are required. Protein micro array confirmed to be a fast, easy and sensitive approach and the results that we obtained with the different controls were sufficiently robust to validate data obtained with the biological samples. As a further confirmation, the different MFIs obtained with either positive or negative sera and which were indicative of differential antigen-specific IgG levels, appeared to be fully consistent with the corresponding IgG titers determined by using a more conventional ELISA assay (Figure 3), confirming what previously observed by Robinson et al. [14], who not only demonstrated that chip and ELISA results correlate but who also highlighted the higher sensitivity of the array approach.

Two major conclusions can be derived from our protein array results. The first one is that the serological profiles observed in tic patients were similar to those observed in sera of patients who experienced a common acute pharyngitis. In fact our analysis established that 46 antigens, out of the 102 present on the chip, reacted against tic and pharyngitis patient sera in a similar manner, being each of them recognized by comparable percentages of sera from the two groups (Tables 1 and 2). Remarkably, the profiles of both sera groups were significantly different from those observed in no tic patients (Table 2 and Figure 2).

The second conclusion coming from the array results is that the IgG response of tic sera appeared to be overall quantitatively stronger than that observed in pharyngitis patients. In fact, when we took into account the frequencies of highly reacting antigens and sera (MFIs higher than 40,000), they appeared to be significantly higher in tic patients, compared to both pharyngitis and no tic patients (Figure 4B and 4C).

Overall, the results discussed so far demonstrated that a large number of GAS antigens eliciting an immune response in the course of a common acute pharyngitis were also recognized, and even more robustly, by tic sera. These data provide the first evidence that tic patients exhibit serological profiles typical of individuals who have mounted a broad, specific and strong immune response against Group A *Streptococcus* antigens, strengthening the relationship between tic disorder and GAS infection, so far based only on discordant epidemiological reports and few signs of infection [15].

The established association between tics and GAS infections raises the question of how and to what extent may the pathogen contribute to the onset or recurrence of this disorder. This is even more intriguing considering that the immune response against GAS antigens which we observed occurred in tic patients in the presence of neuropsychiatric symptoms but in the absence of overt GAS infection, as testified by the lack of clinical signs of pharyngitis and by the usual GAS carrier frequency observed in the tic patient population. Additionally, the fact that no major differences were observed between tic patients with low or high ASO titers further suggested that the serological profiles of these patients were not strictly correlated with the immune response against SLO, which is the parameter routinely used to confirm an ongoing or recent GAS-induced pharyngitis.

Still much has to be explained on how GAS infections contribute to the wide spectrum of post-streptococcal syndrome of the central nervous system (CNS) [16]. It is assumed that CNS disease predisposition is dependent on a multiplicity of factors, among which genetic background [16], [17], and that GAS infections “per se” are not the primary cause of the outcome of CNS disorders but contribute as cofactors triggering the disorder outcome or increasing the risk for the disease to occur, especially in the presence of a particular genetic predisposition.

Induction of non-suppurative sequelae due to “molecular mimicry” between bacterial antigens and host tissues distinguishes Group A Streptococcus from related streptococci and most of other human bacterial pathogens. Protein M is the prototype antigen causing “autoimmune sequelae”, primarily represented by acute RF and by rheumatic heart disease (RHD). These pathologies have indeed been linked to mimicry between M protein and cardiac myosin, due to the immunological response to GAS infection which would cause induction of specific cross-reacting antibodies and inflammatory T cells infiltrating and damaging the myocardium or valve [18], [19]. Similar structural and immunological similarities have been found between M protein and several other auto antigens such as tropomyosin, vimentin, keratin and laminin [1]. The glycolytic enolase enzyme has also been identified as an additional antigen, possibly playing a role in acute RF development. In fact, antistreptococcal enolase antibodies appeared to cross-react with human enolase and sera from acute RF patients had higher anti-human and anti-bacterial enolase titers compared to those found in sera of both pharyngitis and healthy control subjects [20]. Similarly, antibody titers against 5 streptococcal antigens and 4 tissue antigens possibly involved in molecular mimicry, were recently shown to be significantly higher in acute RF compared to pharyngitis patients [21].

The possibility that post-streptococcal autoimmune events similar to those mentioned above could play a role in causing or contributing to neuropsychiatric disorders is still under evaluation. This hypothesis appeared to be supported by the fact that higher levels of antibodies directed against brains structures were observed in children with OCD and PANDAS [22], [23] and that patients with Tourette's syndrome and tic disorder have increased titers of antibodies specific for streptococcal M protein [24], which is known to elicit antibodies cross-reacting with human brains proteins [25]. Further support to the autoimmune hypothesis derived from the observations that monoclonal antibodies obtained from Sydenham's chorea patients [26] showed specificity for mammalian lysoganglioside and N-acetyl-β-D- glucosamine (GlcNAc), the dominant epitope of group A streptococcal carbohydrate, as well as from data indicating the M1 isoform of pyruvate kinase [27] and additional neuronal surface glycolytic enzymes [28] as autoimmune targets in Tourette syndrome and other CNS diseases.

On the basis of this background, our data demonstrating a strong immune response against GAS antigens in tic patients even in the absence of evident infection, suggest that the serological profile observed in these patients may be relevant in the context of one of the current hypothesis [23], [29] proposing that antibodies directed against specific streptococcal antigens could be responsible for auto-immune reactions triggering the occurrence of tic disorders in susceptible individuals. In this scenario, peaks of autoimmune response against critical GAS antigens may occur cyclically, due to repeated boosts, as a consequence of either recurrent infections or re-exposure to the pathogen capable of surviving within the host, causing periodic release and exacerbation of neuropsychiatric or behavioral symptoms in children.

In conclusion, we believe that our data demonstrated the link existing between tic disorder and GAS infection and strengthened the concept that a relationship may exist between tics and antibody response against Group A Streptococcus antigens. While it is presently difficult to evaluate whether an immune response against any of the 51 antigens recognized by tic sera plays indeed a role, we think that these observations could encourage further experiments aimed at better defining the role which specific streptococcal proteins play in the manifestation of children neuropsychiatric disorders.

## Materials and Methods

### Selection, expression and purification of GAS surface-exposed antigens

Computer programs included in the GCG Wisconsin Package version 11.1, in combination with PSORT program, were used to analyze the SF370 strain sequence and to select a subset of predicted surface-exposed and secreted proteins. Proteins included in the subset were those containing leader peptides, lipoprotein signature, outer membrane anchoring motives, host cell binding domains such as RGD and homologies to known surface proteins or to known virulence factors. As a result of this activity, 96 genes were selected from SF370, cloned and expressed in E. coli both as a C-terminal His-tag fusion protein or as a double fusion protein with an N-terminal GST peptide and a C-terminal His-tag [30]. Following IMAC or Glutathione-sepharose affinity-chromatography, the antigens were successfully purified from the bacterial soluble fraction, dialyzed against PBS buffer and used to assemble the GAS-specific protein array. In addition 6 *emm* genes were cloned from strains with different M types and the corresponding proteins were purified and spotted on the chip. The proteins were: M1 from SF370, M2 from 2726 strain, M3 from MGAS315, M9 from 2720 strain, M12 from 2728 strains and M23 from DSM2071. All antigens are listed in Supplementary Table S1.

### Protein micro array technology

The GAS protein array was generated by spotting affinity-purified recombinant protein (0.5 mg/ml) in 4 replicates on nitrocellulose-coated slides (FAST slides, Schleicher and Schuell) with the Chipwriter Pro spotter (Biorad), resulting in spots of approximately 150 µm in diameter. As experimental positive controls to assess the sensitivity and reproducibility of the array set up and for data normalization, a curve of human IgG(s) (solutions from 0.008 to 1 mg/ml) was spotted on the arrays in 8 replicates and detected with Cy3 conjugated α-human IgG secondary antibody. Similarly, a curve of human IgM(s) was also spotted on the arrays, which were undetectable under tested conditions. PBS buffer was spotted in at least twice the number of the protein spots, and used to assess the possible non-specific signal due to cross contamination. For experiments with human sera, slides were saturated with blocking solution 3% Top Block (Fluka-BioChemiKa) −0,1% Tween 20 in PBS (TPBS), and later incubated with sera (1∶1000 dilutions in TPBS) for 1 h at room temperature. After three washes for 5 min in 0.1% TPBS, slides were incubated with Cy3 conjugated anti-human IgG (Southern Biotech) for 1 h at room temperature in the dark. Afterwards arrays were washed twice with TPBS, once with PBS and finally with milliQ sterile water (30 seconds) and were then dried at 37°C for 10–20 minutes in the dark and scanned. Each serum was tested at least twice. Spotting was validated by confirming the presence of all immobilized proteins using mouse anti-GST and anti-His6 tags monoclonal antibodies, followed by detection with a Cy3 labeled α-mouse IgG secondary antibody. Fluorescence signals were detected by using a ScanArray 5000 Unit (Packard, Billerica, MA, USA) and the 16-bit images images were generated with ScanArrayTM software at 10 µm per pixel resolution and processed using ImaGene 6.0 software (Biodiscovery Inc, CA, USA). Elaboration and analysis of image raw fluorescence Intensity (FI) data was performed using an in house-developed software. For each sample, the mean fluorescence intensity (MFI) of replicated spots was determined, after subtraction of the background value surrounding each spot, and subsequently normalized on the basis of the human IgG curve to allow comparison of data from different experiments. The MFIs values of IgG, spotted at different concentrations, were best fitted by a curve belonging to sigmoid family using a maximum likelihood estimator [31]. The normalization method has been set up by defining a reference IgG curve that covers the entire 16 bits pixel range, adjusting the experimental IgG curve and normalizing protein MFIs values accordingly. With the following formula we get the normalized MFI value from the experimental MFI value (Reguzzi V., personal communication):
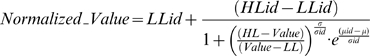
where HLid, LLid, σid and μid are the parameters for the reference sigmoid and HL, LL, σid and μid are the parameters for experimental IgG curve.

In order to profile the specificity of the antigenic responses of each serum and to define the antigen recognition pattern of the three classes of sera, the normalized MFI values were subjected to unsupervised bi-dimensional hierarchical clustering using TIGR Multiexperiment Viewer (MeV) software (http://www.tigr.org/software/tm4/mev.html).

### Human sera

“Pharyngitis” sera were collected from 249 male and female patients aged 4–14 with clinical symptoms of pharyngitis. A throat swab was performed at diagnosis which confirmed pharyngitis being GAS-associated and allowed the isolation of the GAS infective strain, which was then serotyped according to the sequence of the specific M protein antigen. Among the isolated strains, all of the epidemiologically most important M types were represented.

“Tic” sera were collected from patients affected by tic disorders during their first observation at outpatient division of The Department of Child and Adolescent Neuropsychiatry of the University of Rome La Sapienza between September 2004 and July 2005. Inclusion criteria comprised to be affected by tic disorders; exclusion criteria comprised mental retardation, pervasive developmental disorders, tics secondary to neurodegenerative diseases or structural brain lesions of vascular, traumatic or inflammatory origin; prior assumption of drugs or toxins potentially causing secondary tics; children with proved immunologic deficits; non ambulant subjects; institutionalized children. In total, 61 consecutive patients (6 females) affected by tic disorder aged 4–14 years (mean: 9,6; median: 9,2) were studied. All patients were coming from Rome and its province and were attending normal schools. Seventeen patients were recruited during fall, 20 during winter, 18 during spring and 8 during summer. In the large majority of cases the observation occurred during a period of symptom exacerbation in subjects having had tics for months or years; only 3 patients were examined at the onset of tic symptoms. In these patients the severity of tics, as measured by the Yale Global Tic Severity Scale, ranged between 15 and 35 (without impairment score). No patient showed clinical signs of pharingo-tonsillitis; 9 patients (14.75%) had a pharyngeal swab positive for GAS. All isolates were typed according to the sequence of the M protein antigen (*emm* typing). The types identified covered several M types with no prevalence of any specific type. Regarding anti-streptolysin O (ASO) titers, 14 patients showed normal values (<200 International Units, I.U.), 16 intermediate values (200–400 I.U.), 19 elevated values (400–1,000 I.U.) and 12 very elevated values (>1,000 I.U.) No direct correlation by swab positivity for GAS and ASO titer was observed but the mean value for GAS positive patients was in the range that we classified as elevated (about 650 IU). In all, 50.81% of the patients had ASO titers elevated or very elevated. The season of recruitment of patients was not correlated with ASO titer values or with swab positivity for GAS.

“No tic” sera were collected from 35 outpatients (4 females) aged 4–14 years (mean: 8,9; median: 9,1) affected by benign epilepsies attending to The Department of Child and Adolescent Neuropsychiatry of the University of Rome La Sapienza. All patients were coming from Rome or its province and were attending normal schools. The controls were matched to the tic patients for sex, age and time (season of the year) of observation. No patient had tic symptoms or pharingo-tonsillitis. Regarding ASO titer, 16 patients showed normal values (<200 I.U.), 15 intermediate values (200–400 I.U.) and then 4 elevated values (400–1000 I.U.). In all 11.42% of the patients had elevated, and none had very elevated ASO titers.

Human sera were not obtained specifically for this study but were residuals of routine samples collected for diagnosis purposes, which were then used for this study. The Ethics Committee of Policlinico Umberto I°, Università La Sapienza, and the Institutional Review Board of the Methodist Hospital Research Institute authorized the use of residual materials for research purposes. No formal consent was required to perform protein chip experiments since sera and data were used and analyzed anonymously.

### Statistical analysis

Statistical differences among sera groups, ELISA titers and MFIs were analyzed using the two-tailed χ^2^, the T student or the Fisher's exact tests. Differences were considered statistically significant when P values below 0.05 were obtained.

## Supporting Information

Figure S1 GAS antigens preferentially reacting against tic sera. Box and Whiskers plot analysis of Mean Fluorescence Intensity (MFI) values of all Tic, No Tic and Pharyngitis tested sera against the 5 antigens reported in Table III. Medians and extreme values (black dots) are shown.
(0.40 MB TIF)

Table S1 In silico selected GAS antigens
(0.09 MB DOC)
