# Supplementary figures and images for: Protein Array Profiling of Tic Patient Sera Reveals a Broad Range and Enhanced Immune Response against Group A Streptococcus Antigens
Source: PLoS One. 2009 Jul 22;4(7):e6332. doi: 10.1371/journal.pone.0006332 (PMC2709431; doi:10.1371/journal.pone.0006332)

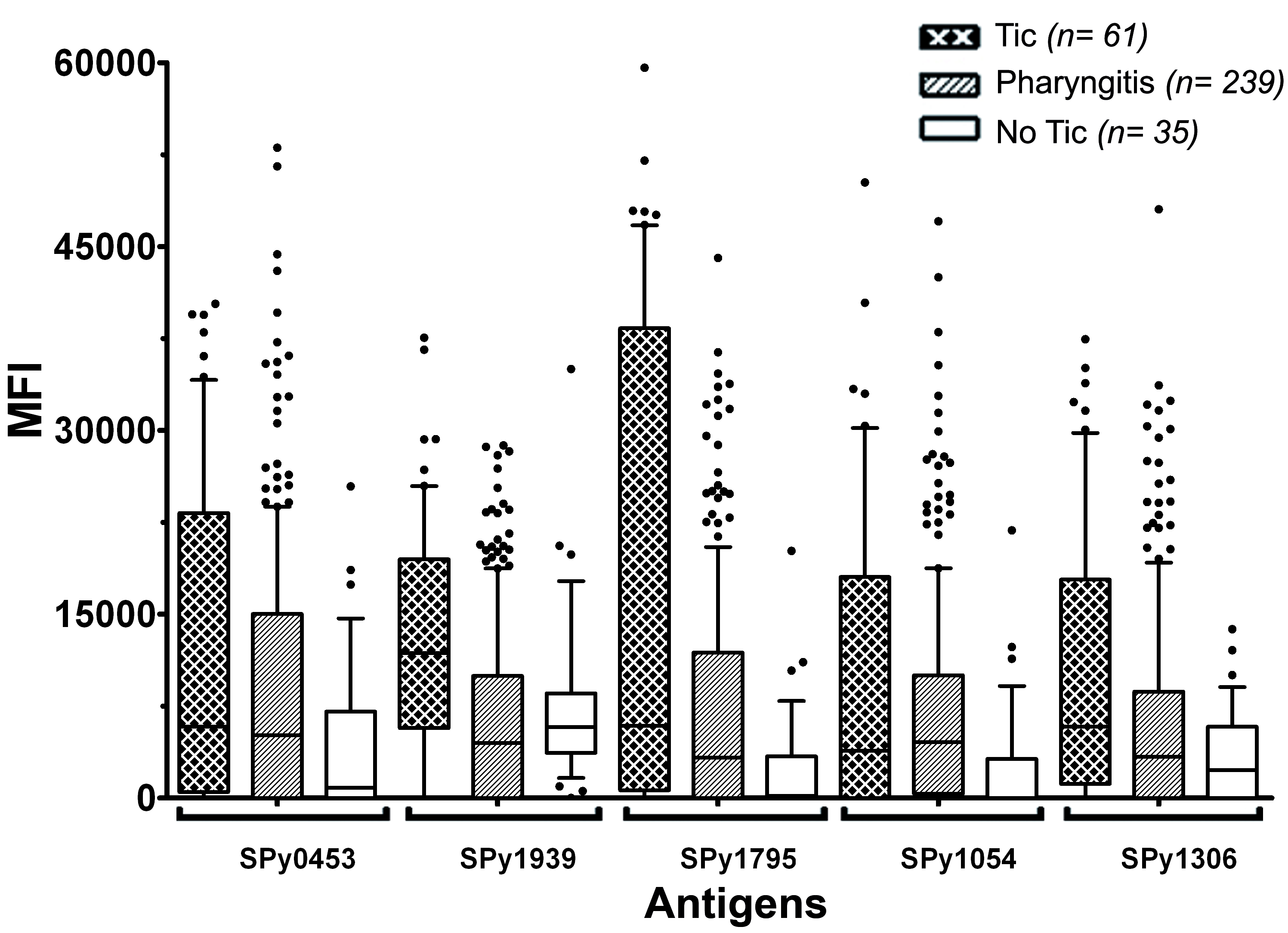

Supplement: Figure S1 — GAS antigens preferentially reacting against tic sera. Box and Whiskers plot analysis of Mean Fluorescence Intensity (MFI) values of all Tic, No Tic and Pharyngitis tested sera against the 5 antigens reported in Table III. Medians and extreme values (black dots) are shown. (0.40 MB TIF) [file pone.0006332.s001.tif]
