# Supplementary material for: Protein Array Profiling of Tic Patient Sera Reveals a Broad Range and Enhanced Immune Response against Group A Streptococcus Antigens
Source: PLoS One. 2009 Jul 22;4(7):e6332. doi: 10.1371/journal.pone.0006332 (PMC2709431; doi:10.1371/journal.pone.0006332)
Supplement: Table S1 — In silico selected GAS antigens (0.09 MB DOC) [file pone.0006332.s002.doc]

## Supplementary Table 1. *In silico* selected GAS antigens

| **SPy ^(a)^** | **Annotation** |
| --- | --- |
| gi-126660 | M protein type 12 |
| gi-4586375 | M protein type 23 |
| gi-507127 | M protein type 9 |
| MGAS10270_SPy1784 | M protein type 2 |
| M5005_SPy_0107^(b)^ | collagen binding protein |
| SPy0019 | putative secreted protein |
| SPy0031 | putative choline binding protein |
| SPy0128 | hypothetical protein |
| SPy0130 | hypothetical protein |
| SPy0159 | hypothetical protein |
| SPy0163 | putative ABC transporter (lipoprotein) |
| SPy0167 | streptolysin O precursor |
| SPy0186 | hypothetical protein |
| SPy0210 | hypothetical protein |
| SPy0212 | exotoxin G precursor |
| SPy0252 | putative sugar transporter sugar binding lipoprotein |
| SPy0269 | putative surface exclusion protein |
| SPy0287 | hypothetical protein |
| M5005_SPy0249^(b)^ | oligopeptidepermease OppA |
| SPy0317 | hypothetical protein |
| SPy0380 | putative manganese-dependent inorganic pyrophosphatase |
| SPy0385 | ferrichrome ABC transporter (ferrichrome-binding protein) |
| SPy0416 | putative cell envelope proteinase |
| SPy0436 | putative exotoxin (superantigen) |
| SPy0441 | hypothetical protein |
| SPy0453 | metal binding protein of ABC transporter (lipoprotein) |
| SPy0457 | putative cyclophilin-type protein |
| SPy0513 | putative XAA-PRO dipeptidase; X-PRO dipeptidase |
| SPy0591 | putative protease |
| SPy0604 | hypothetical protein |
| SPy0652 | hypothetical protein |
| SPy0711 | pyrogenic exotoxin C precursor, phage associated |
| SPy0712 | putative DNase (similar to mitogenic factor), phage associated |
| SPy0714 | putative adhesion protein |
| SPy0731 | phosphopyruvate hydratase (enolase) |
| SPy0737 | putative extracellular matrix binding protein |
| SPy0740 | streptolysin S associated ORF |
| SPy0747 | hypothetical protein |
| SPy0772 | hypothetical protein |
| SPy0778 | putative ABC transporter (substrate-binding protein) |
| SPy0793 | hypothetical protein |
| SPy0838 | hypothetical protein |
| SPy0843 | hypothetical protein |
| SPy0857 | putative peptidoglycan hydrolase |
| SPy0925 | putative oxidoreductase |
| SPy1006 | putative lysin - phage associated |
| SPy1007 | streptococcal exotoxin I |
| SPy1013 | putative fibronectin-binding protein-like protein A |
| SPy1032 | extracellular hyaluronate lyase |
| SPy1037 | hypothetical protein |
| SPy1054 | putative collagen-like protein |
| SPy1105 | putative spermidine/putrescine ABC transporter |
| SPy1173 | glucose-inhibited division protein A |
| SPy1204 | bifunctional GMP synthase/glutamine amidotransferase protein |
| SPy1228 | putative lipoprotein |
| SPy1245 | putative phosphate ABC transporter, periplasmic phosphate-binding protein |
| SPy1274 | putative amino acid ABC transporter, periplasmic amino acid-binding protein |
| SPy1280 | D-fructose-6-phosphate amidotransferase |
| SPy1290 | hypothetical protein |
| SPy1294 | putative maltose/maltodextrin-binding protein |
| SPy1306 | maltose/maltodextrin-binding protein |
| SPy1326 | hypothetical protein |
| SPy1357 | protein GRAB (protein G-related alpha 2M-binding protein) |
| SPy1361 | putative internalin A precursor |
| SPy1390 | putative protease maturation protein |
| SPy1436 | putative deoxyribonuclease |
| SPy1491 | hypothetical protein |
| SPy1497 | putative hemolysin |
| SPy1558 | hypothetical protein |
| SPy1577 | 3-dehydroquinate synthase |
| SPy1618 | putative O-acetylserine lyase |
| SPy1633 | hypothetical protein |
| SPy1697 | hypothetical protein |
| SPy1718 | putative esterase |
| SPy1733 | putative transcription regulator |
| SPy1743 | acetyl-CoA carboxylase alpha subunit |
| SPy1751 | putative trans-2-enoyl-ACP reductase II |
| SPy1795 | putative ABC transporter (periplasmic binding protein) |
| SPy1796 | hypothetical protein |
| SPy1801 | immunogenic secreted protein precursor homolog |
| SPy1813 | hypothetical protein |
| SPy1874 | putative glycoprotein endopeptidase |
| SPy1877 | putative glutamine synthetase |
| SPy1882 | putative acid phosphatase |
| SPy1939 | hypothetical protein |
| SPy1959 | hypothetical protein |
| SPy1972 | putative pullulanase |
| SPy1979 | streptokinase A precursor |
| SPy1983 | collagen-like surface protein |
| SPy2000 | surface lipoprotein |
| SPy2001 | transmembrane transport protein |
| SPy2007 | putative laminin adhesion |
| SPy2009 | hypothetical protein |
| SPy2010 | C5A peptidase precursor |
| SPy2018 | M protein type 1 |
| SPy2025 | immunogenic secreted protein precursor |
| SPy2033 | hypothetical protein |
| SPy2037 | peptidylprolyl isomerase |
| SPy2043 | mitogenic factor |
| SPy2066 | putative dipeptidase |
| SPy2209 | hypothetical protein |
| SPyM3_1727 | M protein type 3 |

^(a)^ when the SPy number is not available, the gi- number is indicated

^(b)^ M5005 SPy number used since the protein was not annotated in SF370, although present
